# Structural genomics of human proteins – target selection and generation of a public catalogue of expression clones

**DOI:** 10.1186/1475-2859-4-21

**Published:** 2005-07-05

**Authors:** Konrad Büssow, Christoph Scheich, Volker Sievert, Ulrich Harttig, Jörg Schultz, Bernd Simon, Peer Bork, Hans Lehrach, Udo Heinemann

**Affiliations:** 1 Protein Structure Factory, Heubnerweg 6, 14059 Berlin, Germany; 2 Max-Planck-Institut für Molekulare Genetik, Ihnestr. 73, 14195 Berlin, Germany; 3 EMBL Heidelberg, Meyerhofstr. 1, 69117 Heidelberg, Germany; 4 RZPD German Resource Center for Genome Research GmbH, Heubnerweg 6, 14059 Berlin, Germany; 5 DIFE, Arthur-Scheunert-Allee 114–116, 14558 Nuthetal, Germany; 6 Max-Delbrück-Centrum für Molekulare Medizin, Robert-Rössle-Str. 10, 13092 Berlin, Germany; 7 Institut für Chemie/Kristallographie, Freie Universität, Takustr. 6, 14195 Berlin, Germany; 8 Department of Bioinformatics, University of Würzburg, Biocenter, Am Hubland, 97074 Würzburg, Germany

## Abstract

**Background:**

The availability of suitable recombinant protein is still a major bottleneck in protein structure analysis. The Protein Structure Factory, part of the international structural genomics initiative, targets human proteins for structure determination. It has implemented high throughput procedures for all steps from cloning to structure calculation. This article describes the selection of human target proteins for structure analysis, our high throughput cloning strategy, and the expression of human proteins in *Escherichia coli *host cells.

**Results and Conclusion:**

Protein expression and sequence data of 1414 *E. coli *expression clones representing 537 different proteins are presented. 139 human proteins (18%) could be expressed and purified in soluble form and with the expected size. All *E. coli *expression clones are publicly available to facilitate further functional characterisation of this set of human proteins.

## Background

### The Protein Structure Factory

The Protein Structure Factory (PSF) is a joint endeavour of universities, research institutes and companies from the Berlin area [1,2]. It takes part in the international structural genomics initiative [3,4] and aims at the determination of human protein structures by X-ray diffraction methods and NMR spectroscopy using standardised high-throughput procedures. A complete pipeline has been established for this purpose that comprises cloning, protein expression in small and large scale, biophysical protein characterisation, crystallisation, X-ray diffraction and structure calculation.

It is known that eukaryotic proteins are often difficult to express in *Escherichia coli *[5]. Only a certain fraction of these proteins can be overproduced in *E. coli *in sufficient yield without formation of inclusion body aggregates or proteolytic degradation. Alternative expression systems include cell cultures of various eukaryotic organisms and cell-free, *in vitro *protein expression. These systems have been greatly improved since 1999, when the PSF project was initiated. In the meantime, *E. coli *[5-7] and wheat germ [8]*in vitro *protein synthesis is routinely used by structural genomics projects. At the PSF, yeast expression hosts, *Saccharomyces cerevisiae *and *Pichia pastoris*, were successfully established as alternative systems to *E. coli*, as described in detail previously [9-11]. We will focus here on the results obtained with the *E. coli *expression system.

### *E. coli *strains and vectors

The T7 RNA polymerase-dependent *E. coli *expression vector system (pET-vectors) is a universal system to generate recombinant protein for structural analysis [12]. pET vectors are usually combined with the *E. coli *B strain BL21 and derivatives that are engineered to carry the T7 RNA polymerase gene. These strains, however, have limitations in cloning and stable propagation of the expression constructs. Expression vectors which are regulated by the *lac *operator are independent of the host strain. Recombination-deficient *E. coli *K-12 strains are suitable for cloning because of their high transformation rates and because they allow for stable propagation of recombinant constructs. The strain SCS1 (Stratagene; *hsd*R17(r_K_^- ^m_K_^+^) *recA1 endA1 gyrA96 thi-1 relA1 supE44*) was found to perform well at the PSF in cloning experiments. It grows relatively fast and allows for robust protein expression.

**Figure 1:**
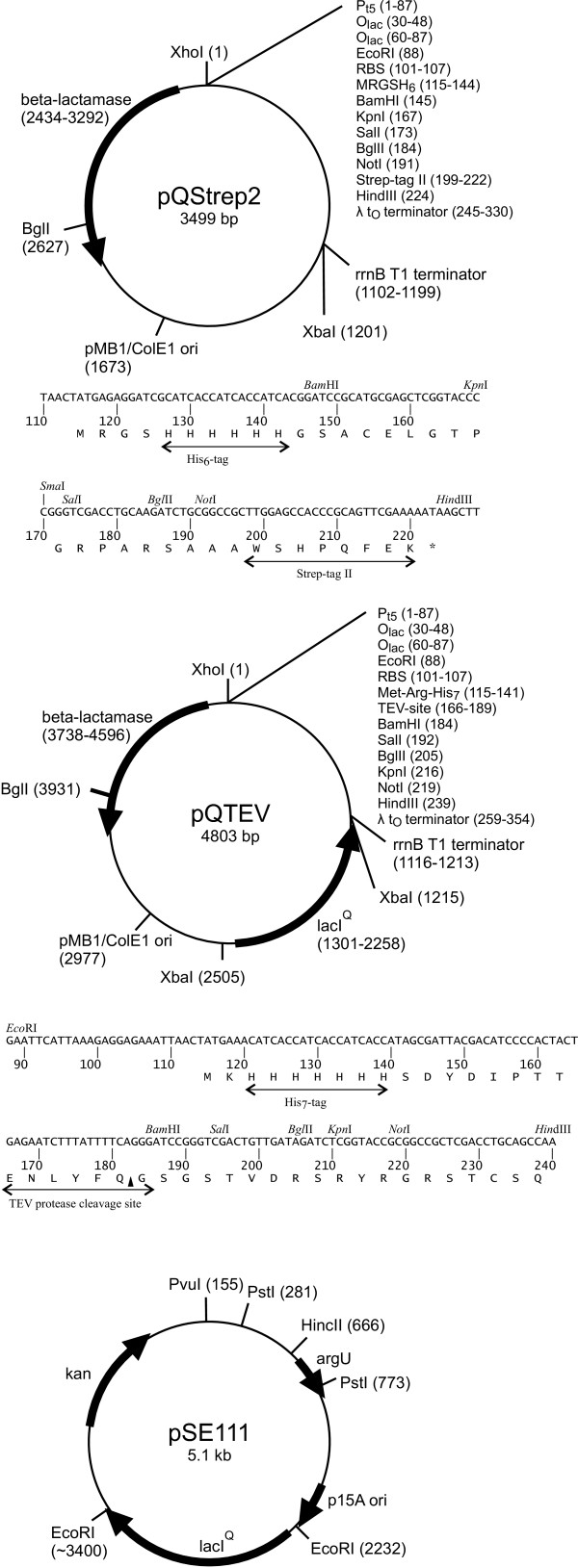
**Vector maps. **Vector maps of pQStrep2, pQTEV and pSE111

Affinity tags allow for standardised protein purification procedures. The first vector that was used routinely in the PSF, pQStrep2 (GenBank AY028642, Figure 1), is based on pQE-30 (Qiagen) and adds an N-terminal His-tag [13] for metal chelate affinity chromatography (IMAC) and a C-terminal Strep-tag II [14,15] to the expression product. pQStrep2 allows for an efficient two-step affinity purification of the encoded protein, as demonstrated in a study of an SH3 domain [16]. The eluate of the initial IMAC is directly loaded onto a Streptactin column. Thereby, only full-length expression products are purified and degradation products are removed. However, the two tags, which are flexible unfolded peptides, remain on the protein and may interfere with protein crystallisation, although we could show that crystal growth may be possible in their presence even for small proteins [16]. To exclude any negative influence by the affinity tags, another vector, pQTEV (GenBank AY243506, Figure 1), was constructed. pQTEV allows for expression of N-terminal His-tag fusion proteins that contain a recognition site of the tobacco etch virus (TEV) protease for proteolytic removal of the tag.

Codon usage has a major influence on protein expression levels in *E. coli *[17], and eukaryotic sequences often contain codons that are rare in *E. coli*. Especially the arginine codons AGA and AGG lead to low protein yield [18]. This can be alleviated by introducing genes for overexpression of the corresponding tRNAs into the *E. coli *host cells. We have used the plasmid pSE111 (Figure 1) carrying the *argU *gene for this purpose. pSE111 is compatible with pQTEV and other common expression vectors. It carries the *lacI*^Q ^gene for overexpression of the Lac repressor, which is required when using promoters regulated by *lac *operators. pSE111 was used at the PSF in combination with the expression vectors pQStrep2 and pGEX-6P-1. Strains for overexpression of rare tRNAs are available from Invitrogen (BL21 Codon Plus) and Novagen (Rosetta). The Rosetta strain contains the chloramphenicol-resistant pRARE plasmid that supplies tRNAs for the codons AUA, AGG, AGA, CUA, CCC, GGA [19]. This plasmid is used at the PSF in combination with pQTEV and pGEX-6P-2.

The Additional file 1 psfClones.xml lists the vector and helper plasmid for overexpression of rare tRNAs that was used for each individual clone.

### Selection of target proteins

We selected target proteins with higher-than-average chances of successful expression in *E. coli *and crystallisation [1]. Proteins were excluded for which sequence analysis predicted that structure determination would be difficult. Starting from the complete set of known human proteins, potentially difficult target proteins and proteins of known structure were excluded according to the following criteria:

• Membrane proteins are known to be complicated targets for structure determination and were excluded. Membrane proteins were identified with the program TMHMM [20,21].

• Since very large proteins are often difficult to express, the maximal length of target proteins was set to 500 amino acids.

• Protein regions that are unstructured or only partially structured [22] may lead to difficulties during protein expression and purification. Unstructured regions are susceptible to proteolyic attack, and represent an obstacle to protein crystallisation. A large proportion of intrinsically unstructured protein sequences are characterised by sequence stretches of low complexity and tandem repeats [23]. Proteins with low complexity regions of more than 20 amino acids length, detected by the SEG program, or with more than one region were excluded [24,25].

• Coiled-coil proteins were excluded from our target list, since this fold is not novel, and structural analysis of coiled coils requires special attention. Many coiled coil proteins form hetero-complexes with other coiled-coil proteins and cannot be studied without their binding partner. Coiled-coils domains are long, extended structures which can usually only be crystallised as domains, i.e. expression constructs lacking other domains have to be prepared. To identify coiled-coil proteins, the program COILS was used [26-28].

• The cellular localisation of target proteins was assigned with the Meta_A(nnotator) [29,30]. Target proteins annotated to be localised in the extracellular space, endoplasmic reticulum, Golgi stack, peroxisome or mitochondria were excluded from expression in *E. coli*. Many of these proteins require formation of disulphide bonds for correct folding, but these are generally not formed in the reducing environment of the *E. coli *cytoplasm. Therefore, these proteins were allocated only for extracellular expression by yeast host cells. Proteins with predicted intracellular localisation or which were not assigned with a localisation by Meta_A(nnotator) were expressed in the cytosol of *E. coli*.

• Potential target proteins were matched to the sequences of proteins with known structure at the Protein Data Bank (PDB) [31]. PSI-BLAST [32] was used to detect even very distinct homologies to PDB entries to rule out proteins with known folds. This filter was later replaced by a less stringent one, which considers the sequence identity and 'coverage' of matches to PDB sequences. The coverage is the length of the sequence match divided by the protein length. According to the less stringent filter, proteins with 60% or more sequence identity over 90% or more of the sequence length were excluded. Thereby target proteins could be included of which only a part, e.g. a single domain, has a known structure.

## Results

### Target lists

The first target list was generated in 1999, when the PSF project started, from the set of all human proteins known at that time. This set was filtered as described above. PSI-BLAST was used to match potential target proteins to the PDB and to include only proteins of presumably novel folds. To enable high-throughput cloning, we selected target proteins for which full-length cDNA clones were available. In 1999, cDNA clones of the IMAGE consortium represented the main public source of sequenced cDNA clones [33]. However, only partial sequence information existed for these clones – the EST sequences of the dbEST database [34] – and only a small proportion contained complete open reading frames (full-ORF clones). 490 proteins were selected which met the selection criteria, had no match to PDB detected by PSI-BLAST and for which full-ORF IMAGE cDNA clones were available.

In 2003, a second target list was compiled from novel full-length cDNAs discovered by the German cDNA consortium [35,36]. The same filter criteria as for the first target list were applied, except that cellular localisation was not taken into account. Proteins with a PDB match of 60% or more sequence identity and 90% sequence coverage were excluded, resulting in a target list of 259 proteins.

A third set of target proteins was selected from a human cDNA expression library (hEx1), which was cloned in a bacterial expression vector. This library was screened for expression clones on high density arrays and by high-throughput protein expression and purification experiments [37-40]. This identified 2,700 clones expressing soluble His-tag fusion proteins that could be purified by affinity chromatography [39]. The cDNA inserts of these clones were sequenced and assigned to sequences of the Ensembl database [41]. 141 proteins represented by clones of the expression library where selected as targets for structural analysis [39]. These clones express soluble, full-length proteins, of which the three-dimensional structure was unknown.

The numbers of targets and success rates grouped by the type of target cDNA clone are summarised in Table 1.

**Table 1:** Origin of template cDNA clones Numbers of targets grouped by type of template cDNA clone

Target list	Number of targets	Number of successfully cloned cDNA	Number of proteins with soluble expression	Number of structures
1 – IMAGE clones	490	264	54	3
2 – cDNA consortium	259	185	34	2
3 – hEx1 library	141	88	51	3

all	890	537	139	8

### Generation and characterisation of expression clones

We established a common cloning strategy that allows for easy shuttling of cDNA fragments between different *E. coli *and yeast vectors. We adopted a cloning system that adds only a minimal number of extra amino acids to the protein of interest and therefore decided to clone with restriction enzymes instead of using alternative systems, such as Invitrogen's Gateway system or ligation independent cloning [42].

The PSF has been working with more than a thousand target proteins to date. Suitable cDNA clones were selected and subcloned into the *E. coli *expression vectors pQTEV GenBank AY243506 and pQStrep2 AY028642[16]. These vectors provide for an N-terminal His-tag; pQStrep2 also encodes an C-terminal Strep-tag-II. Some proteins have also been expressed as GST fusion proteins using the vectors pGEX-4T2 or pGEX-6P1 (Amersham Biosciences).

Expression of protein coding genes from multiple transformants per target was tested under multiple conditions. Standardisation and automation was introduced to achieve this throughput. Expression clones were characterised by small scale protein synthesis at different temperatures, 37°C, 30°C and 25°C, in 1 ml volumes in deep-96-well microplates. Proteins were purified in parallel by a pipetting robot, as described previously [43]. 10% of the purified protein eluate from a 1 ml culture was analysed by SDS-PAGE. For each protein expression experiment, the size of the expression product was recorded and the amount of protein was classified into four categories: none, weak, moderate and strong expression. This classification is arbitrary to a certain degree, however, we found it sufficient to select suitable clones for protein production scale-up.

**Figure 2:**
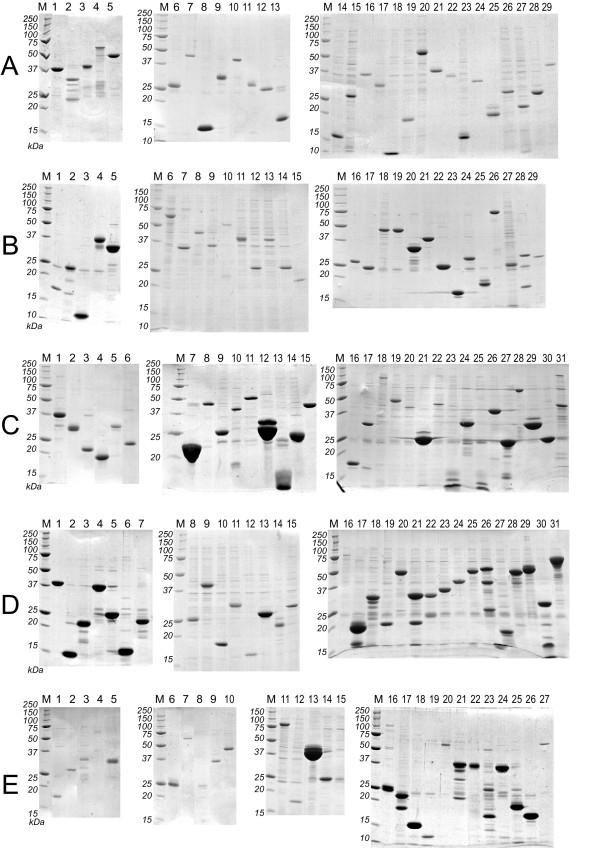
**SDS-PAGE of purified human proteins. **15% SDS-PAGE (Coomassie-stained) of proteins expressed in small scale in *E. coli *and purified by automated immobilised metal chelate affinity chromatography as described in [43]. The identities of the purified proteins are indicated in Table 2. Protein expression was induced at the temperature that is optimal for the individual clone. These temperatures are listed in the supplementary file psfClones.xml. M: Molecular weight marker.

1414 clones for 537 target proteins were successfully cloned in *E. coli *expression vectors, with 473, 191 and 94 target proteins corresponding to target lists one (IMAGE clones), two (DKFZ clones) and three (hEx1 clones), respectively. Clones for 139 different target proteins were found to be expressed in soluble form by *E. coli*. Figure 2 and Table 2 show the result of small scale expression and purification of these proteins. The yield varied significantly among different target proteins. The Additional file 1 psfClones.xml contains further details on the expression clones, such as vector, strain and helper plasmid for overexpression of rare tRNAs.

**Table 2:** PSF *E. coli *expression clones with soluble expression products. The table corresponds to the proteins shown in Figure 2. More detailed information is available in the supplementary XML file, Additional file 1.

NCBI Entrez gene ID	Gene symbol, name	Protein accession	RZPD clone ID	Sequence verified, non-silent mutations	Clone accession	Protein size [Da]	Protein gel, lane
39	ACAT2, acetyl-Coenzyme A acetyltransferase 2	AAM00223	PSFEp250C082	partly, none		44,177	D, 24
203	AK1, adenylate kinase 1	BAA78534	PSFEp250B112	yes, none	DQ000549	24,558	C, 21
689	BTF3, basic transcription factor 3	CAA37376	PSFEp758D0224	no		20,622	A, 27
830	CAPZA2, capping protein (actin filament) muscle Z-line, alpha 2	AAC60382	PSFEp758H1226	no		59,649	A, 4
1036	CDO1, cysteine dioxygenase, type I	BAA12872	PSFEp758D0124	yes, none	DQ000531	25,895	E, 16
1428	CRYM, crystallin, mu	AAC16914	PSFEp758B0810	yes, none	DQ000499	36,275	D, 4
1460	CSNK2B, casein kinase 2, beta polypeptide	CAA34379	PSFEp758H0422	no		26,209	B, 12
1606	DGKA, diacylglycerol kinase, alpha 80kDa	AAC34806	PSFEp758F0324	yes, none	DQ000529	16,408	A, 23
1627	DBN1, drebrin 1	AAH07567	PSFEp250C052	partly, none		74,354	C, 31
1635	DCTD, dCMP deaminase	AAC37579	PSFEp250B121	yes, none	DQ000535	22,938	E, 25
1937	EEF1G, eukaryotic translation elongation factor 1 gamma	AAH15813	PSFEp250H061	partly, none		53,045	C, 11
1974	EIF4A2, eukaryotic translation initiation factor 4A, isoform 2	AAH48105	PSFEp250H052	partly, none		49,301	D, 20
2963	GTF2F2, general transcription factor IIF, polypeptide 2, 30 kDa	CAA42419	PSFEp250B012	yes, none	DQ000547	31,304	C, 17
2992	GYG, glycogenin	AAB00114	PSFEp758H1122	yes, none	DQ000517	40,403	B, 4
3151	HMGN2, high-mobility group nucleosomal binding domain 2	AAA52678	PSFEp250E102	yes, none	DQ000559	12,314	D, 27
3312	HSPA8, heat shock 70 kDa protein 8	BAB18615	PSFEp250E022	partly, none		56,444	D, 31
3735	KARS, lysyl-tRNA synthetase	AAH04132	PSFEp250D032	partly, A116G		70,976	D, 16
3925	STMN1, stathmin 1/oncoprotein 18	CAC16020	PSFEp250F072	yes, none	DQ000556	20,225	D, 19
4043	LRPAP1, low density lipoprotein receptor-related protein associated protein 1	AAC67373	PSFEp250G031	yes, none	DQ000540	44,391	C, 22
4695	NDUFA2, NADH dehydrogenase (ubiquinone) 1 alpha subcomplex, 2	AAD27762	PSFEp250D091	no		13,843	C, 13
4698	NDUFA5, NADH dehydrogenase (ubiquinone) 1 alpha subcomplex, 5	AAD21526	PSFEp250A0510	no		16,381	A, 13
5184	PEPD, peptidase D	AAH28295	PSFEp250H122	yes, none	DQ000560		D, 29
5202	PFDN2, prefoldin 2	AAF17218	PSFEp250D112	yes, none	DQ000555	19,570	D, 17
5412	UBL3, ubiquitin-like 3	AAD02323	PSFEp758A0510	yes, none	DQ000501	15,657	
5502	PPP1R1A, protein phosphatase 1, regulatory (inhibitor) subunit 1A	AAB02402	PSFEp758G0124	yes, none	DQ000526	21,862	A, 15
5716	PSMD10, proteasome (prosome, macropain) 26S subunit, non-ATPase, 10 (gankyrin)	AAH11960	PSFEp250A062	yes, none	DQ000544	27,351	C, 9
5717	PSMD11, proteasome (prosome, macropain) 26S subunit, non-ATPase, 11	AAH04430	PSFEp250B122	yes, none	DQ000548	50,390	C, 19
5877	RABIF, RAB interacting factor	AAB18264	PSFEp250C021	no		16,761	E, 23
6133	RPL9, ribosomal protein L9	BAA03401	PSFEp758E0124	yes, none	DQ000524	24,786	A, 28
6156	RPL30, ribosomal protein L30	CAA55820	PSFEp758A1113	yes, none	DQ000503	15,284	D, 6
6191	RPS4X, ribosomal protein S4, X-linked	BC007308	PSFEp758A0923	no		28,670	
6342	SCP2, sterol carrier protein 2	AAA03559	PSFEp758C0723	yes, none	DQ000515	16,668	B, 1
6451	SH3BGRL, SH3 domain binding glutamic acid-rich protein like	AAC27445	PSFEp758C0713	no		15,274	D, 2
6728	SRP19, signal recognition particle 19 kDa	CAA31280	PSFEp758E0317	no		41,156	
6888	TALDO1, transaldolase 1	AAB53943	PSFEp758B0711	partly, none		40,040	D, 1
6990	TCTE1L, t-complex-associated-testis-expressed 1-like	AAA57444	PSFEp758D0814	no		38,062	A, 3
6993	TCTEL1, t-complex-associated-testis-expressed 1-like 1	BAA09317	PSFEp758F1123	yes, none	DQ000521	13,719	A, 14
7001	PRDX2, peroxiredoxin 2	AAH03022	PSFEp250A042	yes, none	DQ000546	24,815	C, 30
7178	TPT1, tumor protein, translationally-controlled 1	CAA34200	PSFEp250G011	yes, none	DQ000539	22,518	C, 14
7247	TSN, translin	CAA55341	PSFEp250B111	yes, none	DQ000538	29,106	C, 12
7353	UFD1L, ubiquitin fusion degradation 1-like	N P_005650	PSFEp758G0123	yes, none	DQ000519	41,777	D, 9
7390	UROS, uroporphyrinogen III synthase	AAA60273	PSFEp758G0323	no		29,894	D, 11
7518	XRCC4, X-ray repair complementing defective repair in Chinese hamster cells 4	AAH16314	PSFEp250A033	partly, none		41,211	D, 26
7531	YWHAE, tyrosine 3-monooxygenase/tryptophan 5-monooxygenase activation protein, epsilon	AAH01440	PSFEp250H041	yes, A146G	DQ000541	32,097	C, 24
8125	ANP32A, acidic (leucine-rich) nuclear phosphoprotein 32 family, member A	AAB91548	PSFEp250D092	yes, none	DQ000553	31,509	D, 22
8214	DGCR6, DiGeorge syndrome critical region gene 6	CAA65339	PSFEp758G0324	yes, none	DQ000533	12,111	E, 19
8407	TAGLN2, transgelin 2	BAA04802	PSFEp250F062	yes, none	DQ000550	25,314	C, 27
8544	PIR, pirin	CAA69195	PSFEp758A0213	yes, none	DQ000498	34,613	C, 1
8575	PRKRA, protein kinase, interferon-inducible double stranded RNA dependent activator	CAB66550	PSFEp250D014	yes, none	DQ000578	37,328	E, 9
8677	STX10, syntaxin 10	AAC05087	PSFEp250D102	yes, none	DQ000552	25,369	D, 18
8724	SNX3, sorting nexin 3	AAC16040	PSFEp758A0323	yes, none	DQ000514	20,029	B, 15
8896	G10, maternal G10 transcript	AAH22821	PSFEp250F011	yes, A394G	DQ000536	19,766	C, 16
8926	SNURF, SNRPN upstream reading frame	AAD31391	PSFEp250A0610	no		11,334	C, 25
9049	AIP, aryl hydrocarbon receptor interacting protein	AAB59004	PSFEp758F1024	yes, none	DQ000528	40,589	A, 21
9158	FIBP, fibroblast growth factor (acidic) intracellular binding protein	CAG33030	PSFEp250B022	partly, T602C		44,803	C, 20
9168	TMSB10, thymosin, beta 10	AAB25225	PSFEp758A0124	yes, none	DQ000522	6,292	A, 18
9337	CNOT8, CCR4-NOT transcription complex, subunit 8	AAH17366	PSFEp758E0524	yes, none	DQ000523	36,354	A, 24
9453	GGPS1, geranylgeranyl diphosphate synthase 1	AAH05252	PSFEp250E052	partly, none		37,795	D, 23
9465	AKAP7, A kinase (PRKA) anchor protein 7	AAC39715	PSFEp758E0415	no		34,000	A, 1
9796	PHYHIP, phytanoyl-CoA hydroxylase interacting protein	BAA13402	PSFEp758C1124	yes, T806C	DQ000530	40,497	A, 29
10000	AKT3, v-akt murine thymoma viral oncogene homolog 3	CAB55977	PSFEp250B063	yes, none		56,516	E, 20
10063	COX17, COX17 homolog, cytochrome c oxidase assembly protein (yeast)	AAA98114	PSFEp758D0723	yes, none	DQ000516	8,182	B, 3
10094	ARPC3, actin related protein 2/3 complex, subunit 3	AAB64191	PSFEp758G0811	yes, none	DQ000507	23,047	D, 7
10169	SERF2, small EDRK-rich factor 2	AAC63516	PSFEp250A057	no		9,821	C, 23
10228	STX6, syntaxin 6	AAH09944	PSFEp758G0526	yes, none	DQ000509	32,100	B, 7
10247	HRSP12, heat-responsive protein 12 (14.5 kDa translational inhibitor protein, p14.5)	CAA64670	PSFEp758H0822	yes, none	DQ000518	15,760	D, 12
10290	APEG1, aortic preferentially expressed protein 1	AAH06346	PSFEp250B082	yes, none	DQ000543	15,614	E, 18
10539	TXNL2, thioredoxin-like 2	AAH05289	PSFEp250A022	partly, none		40,357	C, 26
10588	MTHFS, 5,10-methenyltetrahydrofolate synthetase	AAC41945	PSFEp758B0824	no		24,522	D, 15
10589	DRAP1, DR1-associated protein 1 (negative cofactor 2 alpha)	AAH10025	PSFEp250C042	yes, none	DQ000542	25,273	E, 3
10598	AHSA1, AHA1, activator of heat shock 90 kDa protein ATPase homolog 1 (yeast)	AAD09623	PSFEp250B091	no		41,199	C, 15
10606	PAICS, phosphoribosylaminoimidazole carboxylase	AAH19255	PSFEp758G0226	partly, none		50,005	A, 5
10842	C7orf16, chromosome 7 open reading frame 16	AAF03537	PSFEp758F0923	no		19,132	B, 2
10856	RUVBL2, RuvB-like 2 (E. coli)	AAH04531	PSFEp250C072	partly, none		54,083	D, 25
10912	GADD45G, growth arrest and DNA-damage-inducible, gamma	AAC83329	PSFEp758F0111	yes, none	DQ000504	19,621	C, 4
10933	MORF4L1, mortality factor 4 like 1	AAH22845	PSFEp250G051	partly, none		40,155	C, 10
10963	STIP1, stress-induced-phosphoprotein 1	AAA58682	PSFEp250A012	partly, A86C		65,566	C, 28
11140	CDC37, CDC37 cell division cycle 37 homolog (S. cerevisiae)	AAH08793	PSFEp250E042	partly, none		47,394	D, 28
11316	COPE, coatomer protein complex, subunit epsilon	AAH07250	PSFEp758G1126	yes, none	DQ000510	37,406	B, 9
11333	PDAP1, PDGFA associated protein 1	AAH07873	PSFEp250A073	yes, none	DQ000554	23,553	D, 30
11337	GABARAP, GABA(A) receptor-associated protein	AAD02337	PSFEp758H0922	no		15,185	D, 10
11344	PTK9L, PTK9L protein tyrosine kinase 9-like (A6-related protein)	N P_009215	PSFEp758A0127	yes, none	DQ000525	42,473	E, 13
22818	COPZ1, coatomer protein complex, subunit zeta 1	AAD34115	PSFEp758A0812	yes, none	DQ000497	22,698	D, 3
22919	MAPRE1, microtubule-associated protein, RP/EB family, member 1	CAB53072	PSFEp250C112	yes, none	DQ000557	32,923	D, 21
22931	RAB18, RAB18, member RAS oncogene family	CAB66668	PSFEp250C118	no		25,900	B, 27
23589	CARHSP1, calcium regulated heat stable protein 1	AAD25021	PSFEp778D072	no		40,934	
25842	ASF1A, ASF1 anti-silencing function 1 homolog A (S. cerevisiae)	AAD34093	PSFEp758E1224	yes, none	DQ000532	25,891	E, 17
25843	PREI3, preimplantation protein 3	AAD34090	PSFEp758B0812	yes, none	DQ000500	28,532	C, 2
25996	DKFZP566E144, small fragment nuclease	AAD34109	PSFEp758G0423	yes, none	DQ000520	25,021	D, 13
26289	AK5, adenylate kinase 5	AAH12467	PSFEp250G042	yes, 3 bp insertion at 3' end	DQ000558	24,897	E, 14
26353	HSPB8, heat shock 22 kDa protein 8	CAB66870	PSFEp250G125	yes, none	DQ000565	24,527	B, 16
27095	TRAPPC3, trafficking protein particle complex 3	AAB96936	PSFEp758A0513	yes, none	DQ000502	22,774	C, 3
27249	C2orf25, chromosome 2 open reading frame 25	AAD20048	PSFEp758D1124	no		35,864	A, 22
27335	eIF3k, eukaryotic translation initiation factor 3 subunit 12	BAA76626	PSFEp758D0923	no		26,326	D, 8
27430	MAT2B, methionine adenosyltransferase II, beta	CAB66599	PSFEp250A114	yes, none	DQ000575	40,476	B, 21
28970	PTD012, PTD012	CAB66540	PSFEp250B014	yes, none	DQ000586	38,042	E, 24
29789	PTD004, GTP-binding protein PTD004	CAB66481	PSFEp250H073	yes, none	DQ000564	47,609	B, 18
30819	KCNIP2, Kv channel interacting protein 2	CAB66656	PSFEp250G064	yes, none	DQ000562	28,964	E, 5
51001	CGI-12, CGI-12	AAH12995	PSFEp250B092	yes, none	DQ000545	40,832	C, 8
51076	CUTC, cutC copper transporter homolog (E. coli), CGI-32	AAH21105	PSFEp758E1112	yes, none	DQ000505	31,811	C, 5
51078	THAP4, THAP domain containing 4	AAD27745	PSFEp250H116	no		21,550	C, 7
51155	HN1, hematological and neurological expressed 1	AAH01420	PSFEp758G0126	yes, 3 bp deletion at 5' end, G457A	DQ000508	18,937	C, 6
51160	VPS28, vacuolar protein sorting 28 (yeast)	AAF00499	PSFEp758C0323	yes, none	DQ000513	26,692	B, 14
51335	NEUGRIN, mesenchymal stem cell protein DSC92	CAD39160	PSFEp250B084	yes, none	DQ000566	27,342	B, 28
51397	COMMD10, COMM domain containing 10	AAD44489	PSFEp758H0811	yes, none	DQ000506	25,466	D, 5
51433	ANAPC5, anaphase promoting complex subunit 5	BAA76629	PSFEp758E0214	no		47,174	B, 8
51451	LCMT1, leucine carboxyl methyltransferase 1	AAH01214	PSFEp758H0126	partly, none		41,303	B, 11
51534	C6orf55, chromosome 6 open reading frame 55	AAF76210	PSFEp758H0426	partly, none		41,303	B, 13
51629	CGI-69, CGI-69	AAD34064	PSFEp758D0316	no		63,564	B, 6
51678	MPP6, membrane protein, palmitoylated 6	CAB66770	PSFEp250E023	yes, none	DQ000583	64,044	E, 27
54816	SUHW4, suppressor of hairy wing homolog 4 (Drosophila)	CAB66569	PSFEp250G074	yes, none	DQ000587	27,032	E, 22
55255	WDR41, WD repeat domain 41	CAD38853	PSFEp250E105	yes, none	DQ000563	26,313	
55276	PGM2, phosphoglucomutase 2	CAB66640	PSFEp250B113	partly, none		71,239	B, 26
56681	SARA1, SAR1a gene homolog 1 (S. cerevisiae)	CAB66658	PSFEp250G115	yes, none	DQ000570	25,290	B, 17
56911	C21orf7, chromosome 21 open reading frame 7	CAD28500	PSFEp250B086	yes, none	DQ000571	19,032	E, 6
57019	LOC57019, cytokine induced apoptosis inhibitor 1	AAC24311	PSFEp758E0424	yes, none	DQ000534	36,506	E, 21
58485	TRAPPC1, trafficking protein particle complex 1	AAD44697	PSFEp250D071	yes, T350C	DQ000537	19,754	E, 1
64284	RAB17, RAB17, member RAS oncogene family	CAB66580	PSFEp250E095	yes, none	DQ000572	26,398	A, 6
79632	C6orf60, chromosome 6 open reading frame 60	CAB66701	PSFEp250B044	yes, none	DQ000568	34,217	B, 20
79666	PLEKHF2, pleckstrin homology domain containing, family F member 2	CAD39132	PSFEp250D054	yes, A271G	DQ000581	30,721	A, 9
79791	FBXO31, F-box protein 31	CAB66696	PSFEp250F085	yes, none	DQ000585	18,588	E, 12
80347	COASY, Coenzyme A synthase	AAF87955	PSFEp250A053	yes, none	DQ000551	33,147	C, 29
80895	ILKAP, integrin-linked kinase-associated serine/threonine phosphatase 2C	CAB66784	PSFEp250D083	yes, none	DQ000569	45,832	B, 19
81876	RAB1B, RAB1B, member RAS oncogene family	CAB66570	PSFEp250C128	no		25,094	A, 12
81889	DKFZP566J2046, fumarylacetoacetate hydrolase domain containing 1	CAB66654	PSFEp250B074	yes, none	DQ000567	27,766	B, 24
83538	DKFZP434H0115, hypothetical protein DKFZp434H0115	CAB66694	PSFEp250G093	partly, none		79,584	E, 11
83543	C9orf58, chromosome 9 open reading frame 58	CAB66501	PSFEp250B085	yes, none	DQ000573	19,990	B, 25
83667	SESN2, sestrin 2	CAB66486	PSFEp250F043	yes, none	DQ000576	57,420	E, 7
84072	NOHMA, HORMA domain containing protein	CAB66689	PSFEp250F083	yes, none	DQ000561	47,343	E, 4
84324	CIP29, cytokine induced protein 29 kDa	CAC37950	PSFEp758H1026	yes, none	DQ000512	26,594	A, 2
84457	PHYHIPL, phytanoyl-CoA hydroxylase interacting protein-like	CAD39006	PSFEp250A084	yes, none	DQ000579	45,425	E, 10
84557	MAP1LC3A, microtubule-associated protein 1 light chain 3 alpha	CAD38714	PSFEp250E106	yes, none	DQ000584	17,194	E, 26
91603	MGC20398, hypothetical protein MGC20398	BAB70992	PSFEp758H0526	yes, none	DQ000511	44,915	B, 10
94240	EPSTI1, epithelial stromal interaction 1 (breast)	CAD38599	PSFEp250C015	yes, none	DQ000580	25,527	A, 11
112611	RWDD2, RWD domain containing 2	CAB52345	PSFEp758A1024	partly, T709C		35,159	B, 5
118812	C10orf83, chromosome 10 open reading frame 83	CAD38849	PSFEp250H085	yes, none	DQ000574	19,158	B, 23
122060	FLJ30046, hypothetical protein FLJ30046	CAD38891	PSFEp250E015	yes, none	DQ000577	23,468	E, 8
136319	MTPN, myotrophin	CAD38909	PSFEp250D016	yes, none	DQ000582	15,817	A, 7
140856	C20orf79, chromosome 20 open reading frame 79	CAB56175	PSFEp758G0224	yes, G258T	DQ000527	20,585	A, 19

Biophysical properties of proteins which could be expressed in soluble form in *E. coli *were compared against all tested proteins. We found no significant correlation between expression success and either protein length or mean net charge (data not shown). However, when analysing the mean hydrophobicity, we found that hydrophobic proteins are less likely to be expressed in soluble form. Only one of 139 well expressed proteins has a mean hydrophobicity of more than 0.2, while 8% of the other proteins are above this value. This group of proteins does not contain transmembrane helices according to TMHMM, and therefore may represent peripheral membrane proteins with hydrophobic surface regions.

**Figure 3:**
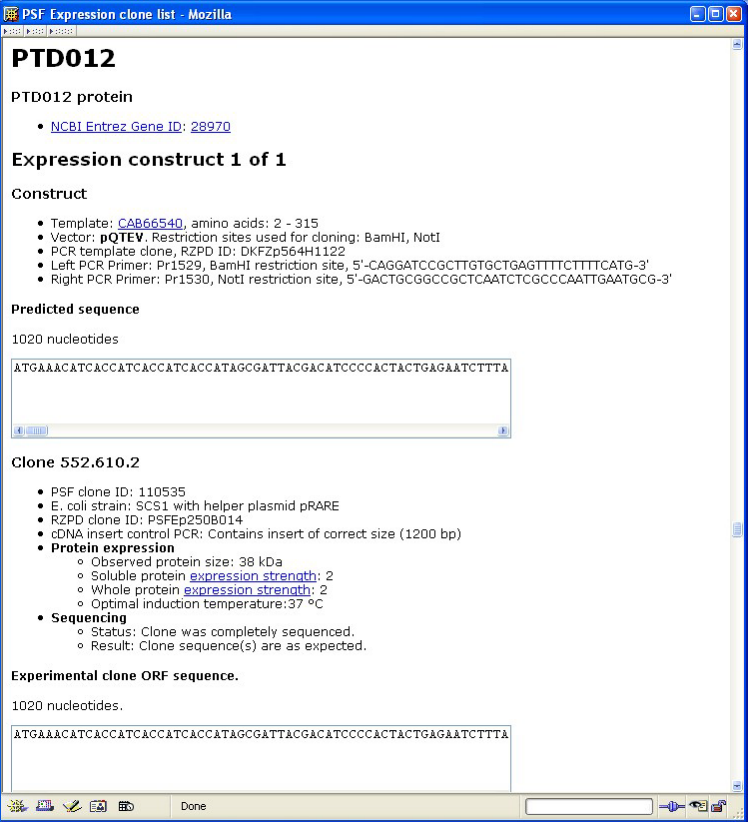
The supplementary XML Additional file 1 file displayed in a web browser.

The *E. coli *expression clones of the PSF are publicly available from the RZPD German Resource Center [44]. The Additional file 1 is an XML list of these clones. It can be viewed in a web browser (Figure 3). The Additional file contains, for each clone:

• Gene ID and name,

• Accession number,

• Cloning details,

• Strain and vector,

• Expected sequence,

• Protein expression results,

• Sequence verification.

### Solved structures

As a result of the target selection and cloning described in this paper, ten novel X-ray structures of human proteins were determined (Table 3). The structure of one protein, TRAPPC3/BET-3, was determined after protein expression in *S. cerevisae*, while the other proteins were produced in *E. coli*.

**Table 3:** Novel human protein structures The structures of full length proteins solved by the Protein Structure Factory.

**NCBI Entrez gene ID**	**Name**	**GenBank protein accession**	**PDB ID**	**Reference**
5716	Gankyrin	AAH11960	1QYM	[55]
10290	APEG1, aortic preferentially expressed protein 1	AAH06346	1U2H	
81889	Fumarylacetoacetate hydrolase family member FLJ36880	CAB66654	1SAW	[56]
28970	PTD012	CAB66540	1XCR	
10247	14.5 kDa translational inhibitor protein, p14.5	CAA64670	1ONI	[57]
27095	BET3, trafficking protein particle subunit	AAB96936	1SZ7	[58]
5184	Peptidase D	AAH28295		
51076	CutC copper transporter homolog, CGI-32	AAH21105		
122553	TPC6	CAI46185	2BJN	[59]
6449	Nicotinamide mononucleotide adenylyltransferase	NP_003012	1GZU	[60]

## Discussion

We describe here the strategies and experiments of our structural genomics project on human proteins. In addition to the expression of full length proteins, the Protein Structure Factory has also studied protein domains by NMR spectroscopy, which has been described elsewhere [45-47]. Our selection of full length target proteins was mainly determined by the availability of full length cDNA clones. In addition, biophysical and bioinformatical criteria were applied, leading to a biased selection of target proteins from the human proteome. Therefore, we expect that the percentage of proteins that we could express and purify in soluble form, 18%, is higher than it would be in a randomly selected set. The low proportion of successfully expressed proteins indicates that *E. coli *is not the appropriate expression host for many full length human proteins. High throughput protein expression in alternative system such as yeast [9-11] or insect cells/baculovirus [48] has been established and will lead to better success rates in future projects.

Generally, clones that did express a soluble protein were verified by DNA sequencing, while clones that did not express or expressed an insoluble product were usually not sequence verified. It cannot be ruled out that some of the unsuccessful clones contain sequence errors introduced during cloning. Since template cDNA clones of the IMAGE consortium with only partial sequence information were used for most cloning experiments, expression clones that were not sequence verified might represent splice variants or isoforms of the original target. The distribution of mean net charge and length was similar among successfully expressed and all proteins, while very hydrophobic proteins were generally not expressed well in our *E. coli *expression system.

Future efforts in structural genomics of mammalian proteins will benefit from a much better supply of full length cDNA clones. Clones prepared for protein expression by resource centres and commercial suppliers are becoming available now. With such resources, alternative target selection strategies will become feasible that will not be restricted by the availability of cDNA clones. Instead, all potential target proteins, including splice variants, could be clustered by similarity and the most suitable members of each cluster could be selected by appropriate criteria as outlined in the Background section.

In our approach, we have excluded certain types of proteins such as membrane proteins and very large proteins. A structural genomics approach that includes membrane proteins would require standard protocols to optimise expression conditions and detergents [49]. The best strategy to study large proteins is to divide them into domains and smaller regions. However, such smaller constructs usually have to be designed manually.

All clones listed in the supplementary file (Additional file 1) and Table 2 are available to the research community. Thereby we hope to facilitate further functional characterisation of this set of human proteins.

## Materials and Methods

### Cloning with restriction enzymes

cDNA inserts were amplified by PCR primers carrying tails with *Bam*HI and *Not*I sites and cloned into the respective sites of one of the expression vectors pQTEV, pQStrep2 or pGEX-6p1. This had the drawback that the restriction sites chosen for cloning might occur in the insert. In such cases, compatible overhangs were produced by alternative enzymes or by the hetero-stagger cloning method [50]. Alternative enzymes are *Bgl*II for *Bam*HI and the type IIs enzymes *Bpi*I, *Eco*31I, *Esp*3I, which can replace both *Bam*HI and *Not*I. Type IIs enzymes cut outside their recognition sequence and can produce arbitrary overhangs.

### PCR Primer design

PCR primers with tails carrying restriction enzyme cleavage sites were designed automatically by a Perl program. The primer design program adjusts the length of the primers to achieve a melting temperature close to a common default. Then, restriction enzymes that do not cut within the respective cDNA sequence are selected by the program and restriction enzyme sites are attached to the primer sequences. Finally, since restriction enzymes do not cut well at the very end of a DNA molecule, an additional short nucleotide tail is automatically attached to the primers. The sequence of this tail is optimised to minimise formation of secondary structure, hairpins or dimerisation. A Java version of the primer design software, 'ORFprimer', is publicly available [51].

### Automated high-throughput cloning, protein expression and purification

PCR primers and cDNA clones were delivered in 96-well microplate format. Upon delivery, plates with PCR primers and template clones were reformatted to obtain corresponding plate positions by a Zinsser Speedy pipetting robot. The PCR master mix (Roche Expand) and cDNA primers (10 μM stocks) were pipetted into a PCR microplate with a multichannel pipet. Template clone bacteria were added with a 96-pin steel replicating device from overnight cultures in microtitre plates. PCR product size and yield was determined by agarose gel electrophoresis and the software Phoretix 1D Quantifier (Nonlinear dynamics). PCR products were purified with magnetic beads on the pipetting robot with a system that has been developed at the Max Planck Institute of Molecular Genetics in collaboration with Bruker Daltonics (Bruker genopure kit). The correct restriction enzyme master mixes were automatically added and the digested fragments were purified again, analysed by agarose gel electrophoresis and quantified. The robot then adjusted DNA concentrations to a common default by dilution. Ligations were set up manually with a multichannel pipet, and SCS1 *E. coli *cells carrying pRARE were transformed in a PCR microplate by chemical transformation on a PCR machine [52]. Transformed cells were manually plated on individual agar plates. Four clones were picked per transformation and were checked by PCR using vector primers. *E. coli *expression clones were ready for protein expression at this stage.

Primer sequences and template clones for cloning of the target cDNAs are listed in the supplementary XML file, Additional file 1.

The characterisation of expression clones by parallel expression and protein purification is described in reference [43].

### Sequence analysis

Sequence analysis software was run with default settings unless indicated otherwise. The mean charge of a protein was calculated as the difference of the number of positive and negatively charged amino acids (Lys, Arg and Glu, Asp, respectively) divided by the protein length. The mean hydrophobicity was calculated with the Kyte and Doolittle hydropathy index [53], obtained from the EMBOSS package [54]. The index values were added up for a given protein and divided by the protein length.

## Authors' contributions

• Study design and coordination: KB, HL, UHe

• Bioinformatics: UHa, BS, JS, PB, VS, KB

• *E. coli *cloning: VS, KB

• *E. coli *protein work: CS, KB

• Manuscript, figures, XML file preparation: KB

## Supplementary Material

Additional File 1 A ZIP archive that contains the XML list psfClones.xml of PSF clones available at the RZPD and the XSL stylesheet psfToHtml.xsl to display the XML file in a web browser. The two files should be extracted from the ZIP archive into a local directory. Then psfClones.xml can be opened with a current web browser like Mozilla or Internet Explorer. 1414 Clones for 537 target proteins are described in the XML file. It lists the gene, cloning details, expected sequence, protein expression results and the degree of sequence verification for each clone. Click here for file
